# Primary orbital melanoma arising in an atypical diffuse (plaque-like) blue naevus/melanocytosis: a case report and review of literature

**DOI:** 10.1186/s12886-021-02176-y

**Published:** 2021-12-09

**Authors:** Tracey-Anne Dickens, Maria Franchina, Adam Gajdatsy, Nima Mesbah Ardakani

**Affiliations:** 1 Centre for Ophthalmology and Visual Science, University of Western Australia, M581, 35 Stirling Highway, Perth, WA 6009 Australia; 2 Lions Eye Institute, 2 Verdun Street, Nedlands, WA 6009 Australia; 3 Department of Anatomical Pathology, PathWest Laboratory Medicine, QEII Medical Centre, Perth, WA 6009 Australia; 4 School of Pathology and Laboratory Medicine, University of Western Australia, 35 Stirling Highway, Perth, WA 6009 Australia; 5 College of Science, Health, Engineering and Education, Murdoch University, Perth, WA 6150 Australia

**Keywords:** Atypical blue naevus, Melanocytosis, Primary orbital melanoma, Case report

## Abstract

**Background:**

Primary orbital melanoma is a rare disease and can occasionally develop from a pre-existing neoplasm of the blue naevus family of melanocytic lesions.

**Case presentation:**

Herein we report a rare case of primary orbital melanoma arising from an unusual atypical diffuse (plaque-like) blue naevus/melanocytosis. A 27 year old man presented with mild pain and swelling of the left eye. Magnetic Resonance Imaging revealed a left lateral episcleral orbital mass and an incisional biopsy confirmed the diagnosis of malignant melanoma. Skin-sparing total left orbital exenteration was performed. Histopathological examination of the exenteration specimen revealed a primary orbital melanoma arising in a pre-existing blue naevus like melanocytosis.

We demonstrate the evidence for histological progression, characterise the molecular profile of this tumour and discuss the related literature.

**Conclusions:**

This case emphasises the importance of a meticulous clinicopathological correlation in recognising such a tumour as a primary orbital melanoma rather than a metastasis, which is managed differently.

## Background

Primary orbital malignant melanoma is a rare tumour. While it can develop de novo, a subset of primary orbital melanomas can arise from a pre-existing melanocytic proliferation either in the form of diffuse melanocytosis or a melanocytic naevus, particularly of the blue naevus family [1–3]. Blue melanocytic naevi, as well as diffuse mucocutaneous melanocytosis (naevus of Ota), are seen not infrequently in the orbital and conjunctival region; however, melanoma arising from such lesions is exceedingly rare and only a handful of cases have been reported [1–18]. Herein we describe the clinicopathological and molecular characteristics of a melanoma arising in an area of unusual and atypical diffuse blue naevus-like melanocytosis and include a review of the relevant literature.

## Case presentation

A 27-year-old Caucasian man presented to the ophthalmology outpatient clinic with mild pain and swelling of his left eye. He had initially presented 18 months previously to the emergency department with an episode of left eye urticaria and spontaneous periorbital bruising, which responded well to topical antihistamine eye drops and lubricants. In addition, for the preceding 6 months the individual had noticed a dark flat lesion on the temporal aspect of his left eye conjunctiva, which was causing a pressure sensation. He had no other significant past medical, surgical or medication history.

**Fig. 1:**
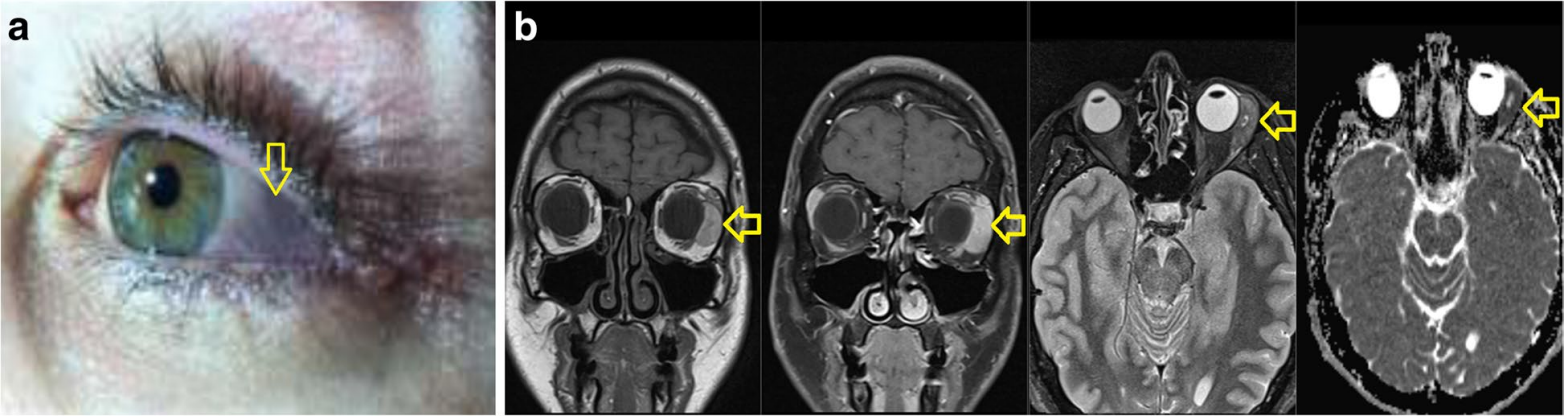
**a** Clinical photo showing an area of blue/grey discoloration and swelling (yellow arrow) involving the temporal bulbar conjunctiva, **b** MRI images from left to right - T1 coronal, T1 coronal with fat saturation and intravenous Gadolinium contrast, T2 Short Tau Inversion Recovery (STIR) axial and Diffusion Apparent Diffusion Coefficient (ADC) highlighting an intrinsically generally T1 hyper-intense, T2 hypo-intense contrast enhancing lateral retro-septal mass (yellow arrows) which exhibits restriction of water movement on diffusion imaging. This is positioned inferior to the lacrimal gland and lateral to the left globe which itself is mildly distorted by the lesion

On examination, an area of grey/blue discoloration associated with swelling was evident at the lateral aspect of left bulbar conjunctiva (Fig. 1a). Visual acuity was normal, the individual had a full range of extraocular movements and there was no proptosis. Dilated fundus examination was normal. An orbital Computed Tomography (CT) scan revealed a 29 mm extraconal left orbital mass involving the lacrimal gland and lateral rectus muscle. Magnetic Resonance Imaging (MRI) of the brain and orbits showed a left lateral episcleral orbital tumour, with extension to the lateral rectus muscle, displacing the lacrimal gland (Fig. 1b). Positron Emission Tomography (PET)/CT scan showed no evidence of an 18-fluorodeoxyglucose (FDG)-avid regional or distant metastasis.

**Fig. 2:**
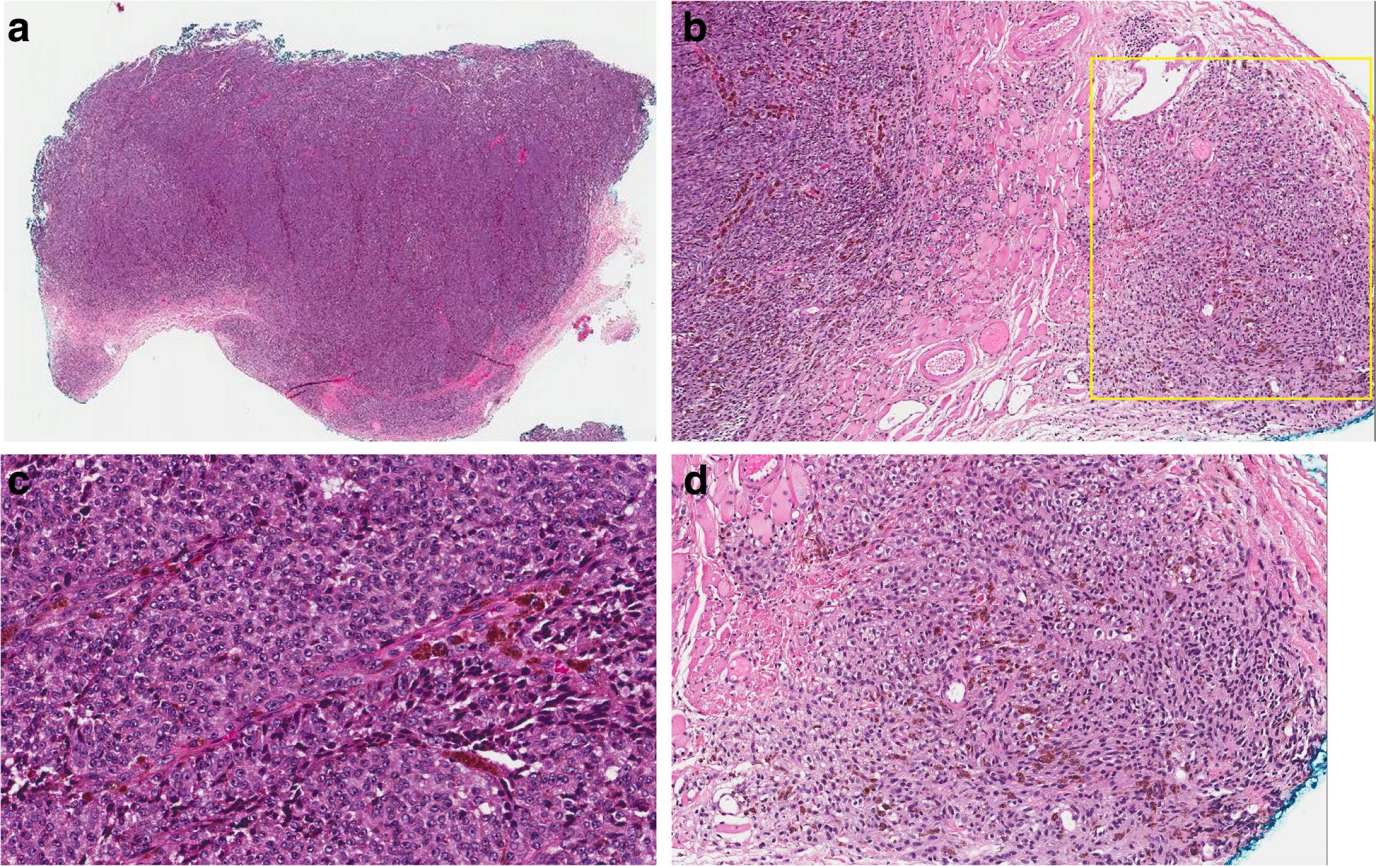
**a** Low power view of a trans-conjunctival incisional biopsy shows portions of a cellular and compacted tumour (H&E, original magnification × 20), **b** invading into the skeletal muscle tissue with an adjacent less cellular lesion on the right side of this microscopic section (yellow rectangle) (H&E, original magnification × 40), **c** the main tumour is composed of densely packed round to oval melanocytes with enlarged pleomorphic nuclei and focal melanin pigmentation (brown pigment in the image), (H&E, original magnification × 200) (**d**) the pre-existing lesion shows features of a blue naevus with loose aggregates of oval to spindle melanocytes with patchy melanin pigmentation (H&E, original magnification × 100)

A biopsy of the lesion located within the lateral rectus muscle was performed via a conjunctival approach. The histopathologic examination showed parts of a malignant melanocytic tumour characterised by compacted sheet-like to fascicular growth of spindled to epithelioid melanocytes showing enlarged pleomorphic nuclei, inconspicuous nucleoli, and moderate eosinophilic cytoplasm. Mitotic figures were easily identified. The tumour was identified as invading the underlying skeletal muscle tissue (Fig. 2 a-c). In addition, at the periphery of this biopsy, there was a distinctly different zone characterised by a component of less atypical melanocytes forming short fascicles and nests of bland appearing oval to spindle to dendritic cells with slightly elongated nuclei, no nucleoli and scant to moderate amount of cytoplasm with focal pigmentation (Fig. 2d). The latter component was suggestive of a pre-existing melanocytic proliferation with histopathological features of a blue naevus family of melanocytic tumours. By immunohistochemistry, the lesional cells demonstrated diffuse positive staining with melanocytic markers including SOX10, Melan-A, S100 and HMB-45. Ki67 proliferation index was estimated to be 5%. There was an abnormal loss of P16 throughout the tumour. BAP1 was retained in tumour cell nuclei.

Molecular testing using Next-Generation sequencing (TruSight 26 gene panel, Illumina, USA) demonstrated a somatic *GNAQ* mutation [c.626A > T, p.(Gln209Leu)], with wild type BRAF and NRAS. Array comparative genomic hybridisation (aCGH; 60 K array, Agilent, Australia) showed whole chromosome 6, 7, 8 and 20 gains. Fluorescence in situ hybridisation (FISH) testing including Melanoma Four-Colour FISH probe (Vysis, Abbott Molecular, USA) was abnormal, showing absolute and relative gain of RREB1.

The overall immunomorphological, molecular and clinical/imaging findings were in keeping with a “primary orbital malignant melanoma arising from a pre-existing blue naevus-like melanocytic proliferation”.

**Fig. 3:**
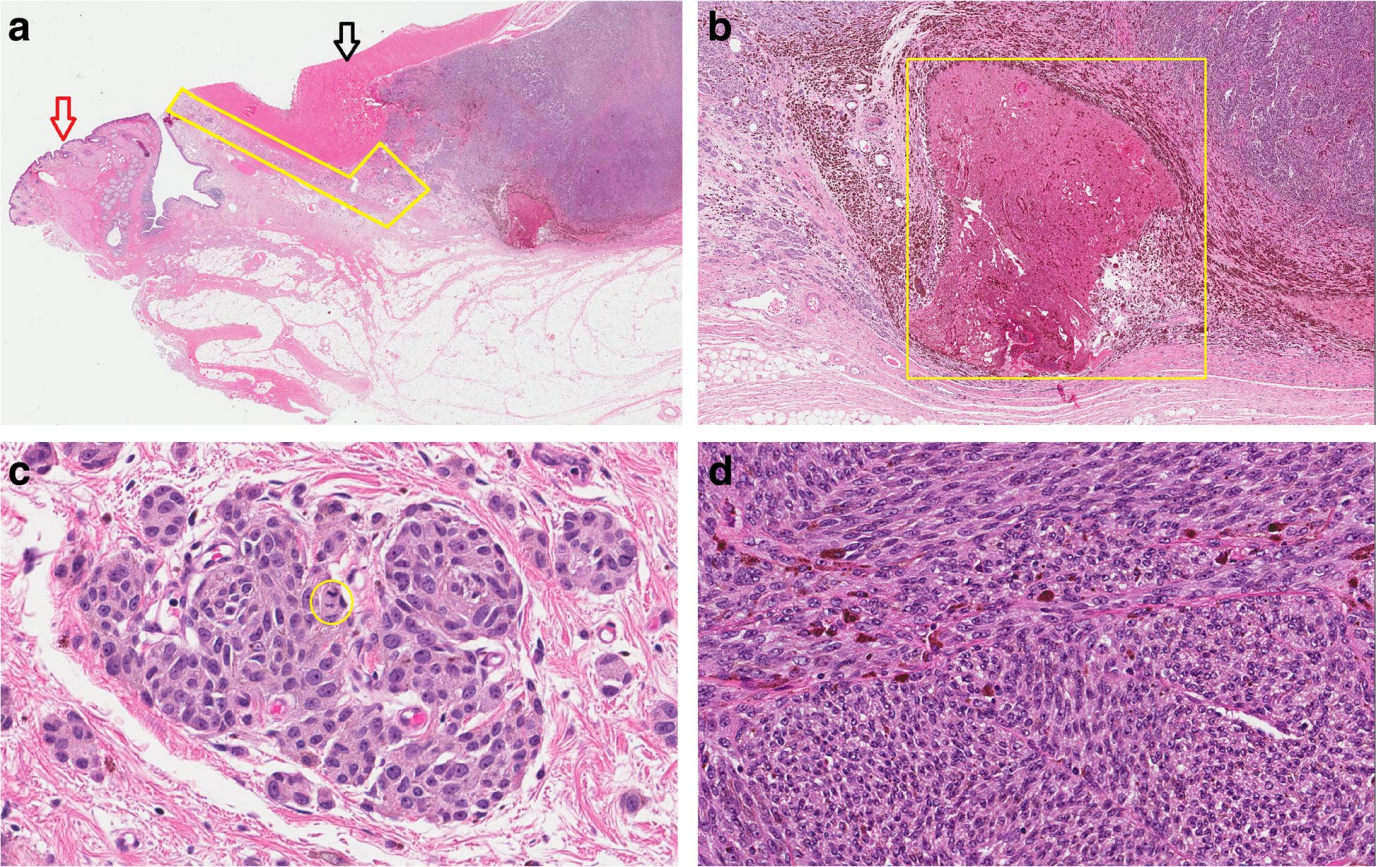
**a** A microscopic section from the exenteration specimen illustrates parts of the eyelid tissue (left, red arrow) and dense fibrous sclera (top, black arrow) with an episcleral/subconjunctival pre-existing melanocytic proliferation (yellow box) giving rise to a densely cellular malignant melanoma (right) (H&E, original magnification × 10), **b** A large area of tumour necrosis (yellow box) is seen at the border between the melanoma (right) and the pre-existing lesion (left) (H&E, original magnification × 30). **c** Microscopic high power view of the pre-existing lesion shows a nested growth of round to oval melanocytes with slightly enlarged nuclei, occasional inconspicuous nucleoli and moderate abundant eosinophilic cytoplasm with occasional mitoses (yellow circle), (H&E, original magnification × 400) (**d**) the malignant melanoma is characterized by compacted fascicular and sheet like growth of highly atypical and mitotically active melanocytes (H&E, original magnification × 200)

The patient subsequently underwent lid skin-sparing total left orbital exenteration. Histopathological examination of the specimen confirmed a malignant melanoma arising in a pre-existing blue naevus-like diffuse melanocytosis. The latter was found to circumferentially involve the sub-epithelial soft tissue of the bulbar conjunctiva as well as episcleral soft tissue toward the temporal aspect of the orbit. The pre-existing lesion was histopathologically characterised by an interrupted rim of clusters and nests of spindle to epithelioid melanocytes with patchy pigmentation, mild to moderate cytologic atypia and low-level mitotic activity (Fig. 3a-b). There was an abrupt transition to an expansile tumour (melanoma) showing compacted sheet-like to fascicular growth of atypical plump melanocytes with frequent mitoses (up to 3–4 mitoses per square millimeter), an area of necrosis and extensive infiltration of the lateral rectus muscle. The immunohistochemical profile of the tumour was identical to the prior incisional biopsy (Fig. 3c-d). The lacrimal gland was intact. The internal orbital structures, including the anterior and posterior uvea as well as the optic nerve, were intact with no evidence of a melanocytic proliferation in the iris, ciliary body or choroid. The histopathological findings in the exenteration specimen confirmed the initial diagnosis of malignant melanoma arising in a pre-existing atypical diffuse plaque-like blue naevus/melanocytosis.

The patient declined post-operative radiotherapy and opted for close surveillance with regular PET and MRI scans every 3 months. As of 26 months’ follow-up, no recurrence or metastasis has been detected thus far.

## Discussion and conclusion

The blue naevus family of melanocytic neoplasms, including several variably termed melanocytoses such as naevus of Ota, naevus of Ito and Mongolian spot, is a heterogenous group of melanocytic proliferations occurring in the skin and soft tissue, meninges, and mucosa. Such lesions, including oculodermal melanocytosis also known as “naevus of Ota”, can develop in the conjunctiva, eyelid, sclera, and peri-orbital tissue. The exact incidence is not clear; however, blue naevi encompassed 1.5% of pigmented and 0.2% of all conjunctival lesions in a large series of 2455 adult patients reported by Grossniklaus and colleagues [19]. Clinically, these neoplasms manifest as variably pigmented, sometimes very dark/blue lesions, which can be flat or slightly raised. Deep-seated lesions located in the subcutis or peri-orbital soft tissue can be clinically undetectable. Histologically, blue naevi and melanocytoses are characterised by variably dense proliferation of elongated to dendritic melanocytes with thin bland spindle nuclei, often with cytoplasmic melanin pigmentation, within a usually sclerotic stroma with different proportions of intermixed melanophages. Histologic variations including cellular and atypical cellular blue naevi have been described, typically presenting as bulbous to dumbbell-shaped proliferation of fascicles of compacted spindled to ovoid melanocytes with a mild degree of cytologic atypia and limited mitotic activity (≤ 2 mitoses/square millimeter) [20]. At the molecular level, blue naevi and mucocutaneous melanocytoses, similar to uveal melanocytic neoplasms, harbour somatic mutations in genes encoding G protein, including *GNAQ* and *GNA11* in most cases, with a minority showing alterations in *PLCB4* or *CYCLTR2* genes as an initiating genomic event [21].

While most melanocytic neoplasms of the blue naevus family follow a benign clinical course, a stepwise malignant transformation due to accumulation of additional genomic alterations can occur rarely. Such a transformation has been histologically documented by transition from overtly benign-appearing foci to atypical areas characterised by increased cellularity, cytologic atypia and occasional mitoses to areas showing a frankly malignant appearance with compacted sheet-like and fascicular growth of melanocytes, with significant cytologic atypia, high mitotic rate (> 2 mitoses/square millimeter) and necrosis [13, 20], as observed in the current case. However, a transition such as this is not always observed, particularly in advanced tumours where the melanoma has completely overrun the pre-existing lesion or developed de novo. At the molecular level, these melanomas often show additional genomic alterations including bi-allelic inactivation of BRCA-associated protein 1 (*BAP-1*) gene or mutations in the *SF3B1* gene [21]. In cytogenetic studies, multiple gains and losses of chromosomes, specifically losses of chromosome 3, have often been reported in melanomas associated with blue naevus [20].

The biologic behaviour of histologically malignant tumours of blue naevus origin (variably termed as melanoma associated with blue naevus, melanoma ex-blue naevus, malignant blue naevus and blue naevus-like melanoma) is comparable with conventional melanoma in which local recurrence, metastatic spread and death from the disease are frequently reported [22]. In a previous study, Martin et al. demonstrated no difference in patient survival and overall clinical behaviour of blue naevus-like melanomas compared with conventional melanomas matched for patient age and sex, Breslow thickness, Clark level, ulceration and anatomical location [23]. In addition, it has been shown that tumour thickness — measured as Breslow thickness, or in the absence of epidermis, the largest dimension of tumour — is the only clinicopathological factor significantly correlating with both shorter recurrence-free patient survival and reduced time to distant metastasis. In contrast, other previously established prognostic factors for conventional melanoma, such as patient age and gender, ulceration, tumour necrosis, lymphovascular invasion and mitotic activity, do not correlate with the outcome in this subset of melanomas [15, 23].

Identifying true cases of melanoma arising in blue melanocytic proliferations in the literature is difficult due to the confusion surrounding terminology and variable inclusion of different entities by different authors. The first report of orbital melanoma arising in association with a blue melanocytic proliferation dates back to 1954 when Dorsey and Montgomery reported two cases of malignant melanoma arising from congenital naevus of Ota, one in a 24-year-old woman who died from metastatic disease 3 years later and the other one in a 16-year-old boy [4]. Following on from this, in 1965, Jay presented a clinicopathologically well-documented case of a 66-year-old woman with progressive right eye proptosis due to a retroocular mass on a background of oculodermal melanocytosis and multiple blue naevi in the subcutaneous tissue of the eyelid [5].

Including our patient, in the English literature to date 27 well-documented cases of primary orbital melanoma arising from the blue naevus family of melanocytic neoplasms have been reported (Table 1). This cohort appears to be mainly composed of patients of Caucasian ethnicity, with an average age of 40 years (median 36, range 9–70 years) with no significant gender predilection (F/M = 1.1). The lesions developed in both periorbital skin and deep periorbital tissues, including the intraconal space, with an average size of 20.7 mm (range 8–30 mm). The clinical presentation ranged from an asymptomatic or slightly painful enlarging mass to progressive proptosis, diplopia, and loss of vision. Histologically, the most common pre-existing lesion was a “cellular blue naevus”, seen in 55.5% of patients, sometimes combined with other proliferations of blue naevus family. A naevus of Ota was present in 8 (⁓ 30%) cases and a component of common blue naevus was seen in 11 (⁓ 41%) cases. The melanoma component in these cases has been reported as a nodular expansion of variably pigmented tightly packed atypical melanocytes, often with mixed spindle and epithelioid morphology and significant cytologic atypia with enlarged pleomorphic nuclei and prominent nucleoli. Increased mitotic activity and necrosis were also common findings in these melanomas [2, 5–7, 9, 10, 13–15] .

**Table 1:** Clinicopathological characteristics and follow up data on primary orbital melanomas arising in blue naevus family of melanocytic proliferations

Case	Gender	Age	Race	Site	Size/ thickness	Clinical Presentation	Associated Lesion	Surgical Management	Histopathology	Genomic Findings	Follow up			Reference	Year
											Time	Consequence	Alive		
1	F	24	N/A	Lateral orbit/temple	N/A	N/A	Naevus of Ota	N/A	Not well described	N/A	36 months	Metastasis (liver)	N	Dorsey [4]	1954
2	M	16	White	Right orbit beneath the zygoma	N/A	Subcutaneous mass	Naevus of Ota and BN	Excisional biopsy	Not well described	N/A	N/A	N/A	N/A	Dorsey [4]	1954
3	F	64	N/A	Right retroorbital	N/A	Progressive proptosis	Naevus of Ota and BN	Exenteration	Expansile nodule of predominantly epithelioid cells with necrosis and haemorrhage	N/A	10 months	Metastasis	N	Jay [5]	1965
4	M	57	White	Left apex of orbit	N/A	Protrusion of eye, diplopia, and gradual loss of vision	Naevus of Ota, BN, CBN	Exenteration	Mixed epithelioid and spindle melanocytes with moderate nuclear pleomorphism, prominent nucleoli and mitotic activity	N/A	10 months	None	Y	Hagler [6]	1965
5	F	29	White	Right floor of orbit	N/A	Protrusion of eye and diplopia	Naevus of Ota, BN, CBN	Partial exenteration	Predominantly spindle cells with moderate pleomorphism, mitotic activity and necrosis	N/A	24 months	Metastasis (liver)	N	Speakman [7]	1973
6	F	42	Hispanic	Left supraorbital	25 mm	Progressive proptosis and diplopia	CBN and Plexiform pigmented neurofibroma	Exenteration	Mainly epithelioid melanocytes, with large nucleated nuclei and low mitotic activity	N/A	N/A	N/A	N/A	Jakobiec [8]	1974
7	M	15	White	Left inferonasal	N/A	Lateral displacement of orbit	Naevus of Ota and CBN	Transfrontal craniotomy	Predominantly pleomorphic spindle cells with large pleomorphic nuclei, prominent nucleoli, variable pigmentation and mitotic activity	N/A	18 months	no recurrence	Y	Dutton [9]	1984
8	F	67	White	Left orbit at the site of previous enucleation due to severe end stage unilateral glaucoma	N/A	Mass at the site of previous enucleation	Naevus of Ota		Mixed spindle and epithelioid cells, with moderate nuclear pleomorphism and prominent nucleoli and mitotic activity	N/A	N/A	N/A	N/A	Dutton [9]	1984
9	M	27	N/A	Right orbit posterior and inferonasal	26 mm	Proptosis, pain and exophthalmos	CBN	Exenteration	Expanisle nodule of packed epithelioid to spindle cells with pleomorphic nuclei and prominent nucleoli with mitotic activity and areas of necrosis and haemorrhage	N/A	12 months	None	Y	Loffler [10]	1989
10	M	36	N/A	Eyelid	N/A	N/A	BN	N/A	Not specifically described	N/A	60 months	N/A	N	Connelly [11]	1991
11	F	32	White	Right upper and lower eyelid	N/A	Progressive enlargement and confluence of pigmentations and nodules involving both upper and lower eyelids and inferior conjunctival fornix	Diffuse multifocal CBN	Debulking excision of lower eyelid	Not well described	N/A	132 months	Local recurrent (right orbitopalpepbral) and metastasis (right middle cranial fossa/frontotemporal brain)	N	Gunduz [12]	1998
12	M	29	White	Left inferior anterior orbital/eyelid mass	20 mm	Progressive enlargement of periorbital and lower eyelid blue discoularation with emergence of a subcutaneous mass	CBN	Orbitotomy	Packed cellular nodule of pigmented atypical spindled and epithelioid melanocytes	N/A	132 months	Local recurrence (lower eyelid), metastasis (ipsilateral preauricular lymph node)	Y	Gunduz [12]	1998
13	M	41	White	Left conjunctiva	N/A	Progressive enlargement and confluence of multifocal pigmentations involving the entire inferior fornix and inferior bulbar conjunctiva	BN	Excisional biopsy of conjunctiva	Focal nodular expansion of epithelioid cells with large pleomorphic nuclei with prominent nucleoli	N/A	13 months	None	Y	Demirici [1]	2000
14	M	36	N/A	Inferior orbit	N/A	N/A	BN	N/A	Mixed epithelioid and spindle cells with cytologic atypia and pleomorphism, necrosis and mitotic activity	N/A	36 months	Local recurrence	Y	Granter [2]	2001
15	F	36	White	Right intraconal superomedial orbit	18 mm	Exophthalmos	Naevus unclassified (possibly amelanotic blue naevus based on description)	Exenteration	Mixed epithelioid and spindle cells with moderate nuclear pleomorphism and mitotic activity	N/A	24 months	None	Y	Mandeville [3]	2004
16	F	43	White	Right intraconal space	25 mm	Sudden pain, proptosis and decreased vision	BN	Initial orbitotomy and biopsy, followed by craniotomy and subtotal removal of the tumour (16 months later), and finally exenteration (3 months after craniotomy)	Predominately epithelioid cells with large nuclei and prominent nucleoli with scattered mitoses	N/A	19 months	Local aggressive growth (erosion through the roof of the orbit to the level of dura mater)	Y	Odashiro [18]	2005
17	F	52	White	Left retroorbital	N/A	Rapidly progressive loss of vision in left eye	Naevus of Ota and CBN	Exenteration	Expansile nodular growth of spindle and epithelioid cells with necrosis and prominent mitotic activity	BRAF, GNAQ, NRAS and KIT WT	11 months	None	Y	Gerami [13]	2010
18	F	44	N/A	Right optic nerve	N/A	Intermittent right side visual disturbance and optic disc oedema	CBN	Exenteration	Epithelioid and spindle cells forming an expansile nodule with tumour necrosis	N/A	24 months	None	Y	El-Sawy [14]	2014
19	F	54	N/A	Left orbital mass	N/A	Proptosis of the left eye	CBN	Exenteration	Well circumscribed nodule of monotonous epithelioid cells	N/A	24 months	None	Y	El-Sawy [14]	2014
20	F	9	N/A	Right orbit with extension into pterygopalatine fossa	N/A	Progressive proptosis of right eye and swelling of the eyelid	CBN	Exenteration plus partial maxillectomy, ethmoidectomy and sphenoidotomy	Well circumscribed expansile nodule of monotonous epithelioid cells with increased proliferation index as judged by ki67 immunostain	N/A	36 months	None	Y	El-Sawy [14]	2014
21	F	33	White	Orbit	14 mm	Blurry vison	Atypical CBN	N/A	Epithelioid and spindle cells	N/A	85.2 months	Metastasis (liver)	Y	Loghavi [15]	2014
22	F	54	White	Orbit	8 mm	Exophthalmos	BN	N/A	Epithelioid with necrosis and mitotic activity and PNI	N/A	27.6 months	None	Y	Loghavi [15]	2014
23	M	59	N/A	Left intra- and extraconal orbit with extra orbital extension into pterygopalatine fossa	N/A	Blurred vision, eyelid swelling and blind spot in visual field	CBN	Orbitotomy	Densely hypercellular tumour of spindle and epithelioid melanocytes with vesicular nuclei, prominent nucleoli and focal melanin pigmentation and high proliferation index as judged by Ki67	N/A	N/A	Possible metastasis to the lung on imaging (not proven histologically)	Y	Hussain [16]	2017
24	M	27	N/A	Superior orbit	30 mm	Mass lesion	CBN	Exenteration	Expansile nodule of intensely pigmented atypical spindle and epithelioid melanocytes with hyperchromatic pleomorphic nuclei and increased mitotic activity and atypical mitoses	BRAF V600E negative (IHC), BAP-1 loss (IHC)	N/A	N/A	N/A	Figueira [17]	2018
25	F	70	N/A	Right inferolateral orbit	20 mm	Diplopia and proptosis	BN	Exenteration	A nodule of spindle to ovoid atypical melanocytes with mitotic activity	BRAF V600E negative (IHC), BAP-1 loss (IHC)	N/A	N/A	N/A	Figueira [17]	2018
26	M	50	N/A	Left superolateral orbit	21 mm	Proptosis	CBN	Exenteration	Densely hypercellular and intensely pigmented nodule of spindle and epithelioid melanocytes with hyperchromatic and pleomorphic nuclei increased mitotic activity and atypical mitoses	BRAF V600E negative (IHC), BAP-1 retained (IHC)	N/A	N/A	N/A	Figueira [17]	2018
27	M	27	White	Left lateral/temporal extraconal orbit	29 mm	Mild pain, eye swelling and lateral conjunctival grey/blue discoloration	Atypical diffuse BN like melanocytosis	Exenteration	A nodule of spindle to ovoid atypical melanocytes with mitotic activity	GNAQ mutation, BRAF, NRAS and KIT WT	26 months	None	Y	Current Case	

*N/A* Not Available, *BN* Blue Naevus, *CBN* Cellular Blue Naevus.

While pre-existing lesions in all of the previously reported cases of orbital blue naevus-like melanoma could be easily categorised into the currently known subtypes of blue melanocytic proliferations, the precursor lesion in our case was extraordinary due to the peculiar histologic features including a diffuse circumferential and multifocal architecture and mild cytologic atypia and mitotic activity of constituent melanocytes, albeit with an abrupt transition to a frankly malignant tumour. The diffuse nature of this lesion is akin to what has been previously described as “plaque-like blue naevus” in the skin [24, 25]; however, the atypical cytology and mitotic activity is unusual. Therefore, we opted to classify the lesion descriptively as an “atypical plaque-like blue naevus/melanocytosis” in an orbital location. We are not aware of a similar lesion being described in association with a blue naevus-like melanoma in the orbit.

The molecular profile of these tumours has not been well elucidated and comprehensive molecular testing was available in only two cases, one of which (the current case) showed a *GNAQ* mutation. Molecular or immunohistochemical data on BRAF V600E was available in 5 cases, all of which were wild type [13, 17]. Loss of BAP-1 protein by immunohistochemistry, indicating bi-allelic inactivation of *BAP-1* gene, has been shown in two cases [17]. Although very limited, as expected, the molecular data available suggest that the molecular profile of these tumours is similar to that of uveal melanoma and cutaneous melanoma associated with blue naevus, with frequent initial somatic mutations in G protein-encoding genes followed by bi-allelic inactivation of *BAP-1* through different mechanisms including chromosomal loss as well as somatic or germline mutations in *BAP-1* gene and possibly epigenomic means in some cases [26].

Follow-up was reported for 20 patients, with an average follow-up time of ~ 38 months. While 4 patients developed local recurrences [2, 12, 18], metastases were reported in 7 patients [4, 5, 7, 12, 15, 16], with tumour spreading to the liver (3 patients), lymph node, brain, and lung and 5 patients dying from the disease [4, 5, 7, 11, 12]. These data confirm the speculation that primary orbital melanoma associated with blue naevus, similar to such tumours elsewhere and conventional melanoma, is potentially an aggressive disease requiring appropriate local clearance with close follow-up.

This case also emphasises the importance of differentiating primary orbital melanoma from metastatic orbital melanoma or spread from adjacent tissues as treatment options differ; exenteration is the treatment of choice for primary melanomas, but non-surgical treatment is preferred in the case of metastasis. In our case, the presence of a pre-existing lesion in the form of an atypical plaque-like blue naevus/melanocytosis in an episcleral/submucosal conjunctival location and the absence of evidence of a primary melanoma in the uvea or an extra orbital location (based on PET scan) pointed toward a primary orbital tumour. Given the rarity of such cases, the role of systemic therapy is not clear.

In summary, we report a primary orbital melanoma developing in an unusual pre-existing melanocytic proliferation of blue naevus family, which we descriptively categorised as “atypical plaque-like blue naevus/melanocytosis”. This phenomenon is extremely rare and seems to occur due to accumulation of genomic alterations at the molecular level. The biologic nature and clinical behaviour of such melanomas are comparable to conventional melanomas. This case highlights the need to recognise “melanoma ex-blue naevus” as a primary orbital tumour that mandates careful histologic examination to look for a pre-existing lesion, with comprehensive clinicopathological correlation to avoid misdiagnosing such melanomas as a metastatic tumour, which is managed differently.

## Data Availability

All data generated or analysed during this study are included in this published article.

## Abbreviations

FISH: Fluorescence in situ hybridization
MRI: Magnetic Resonance Imaging
CT: Computed Tomography
PET: Positron Emission Tomography
